# Cellular MicroRNA Expression Profile of Chicken Macrophages Infected with Newcastle Disease Virus Vaccine Strain LaSota

**DOI:** 10.3390/pathogens8030123

**Published:** 2019-08-09

**Authors:** Jiaqi Mu, Xinxin Liu, Xibing Yu, Junjiao Li, Yidong Fei, Zhuang Ding, Renfu Yin

**Affiliations:** 1 Department of Veterinary Preventive Medicine, College of Veterinary Medicine, Jilin University, Xi’an Road 5333, Changchun 130062, China; 2 College of Food Science and Engineering, Jilin University, Xi’an Road 5333, Changchun 130062, China

**Keywords:** Newcastle disease virus, vaccine strain, chicken macrophages, microRNA, LaSota

## Abstract

Vaccines with live, low-virulence Newcastle disease virus (NDV) strains are still the most accepted prevention and control strategies for combating Newcastle disease (ND), a major viral disease that hampers the development of the poultry industry worldwide. However, the mechanism underlying vaccine-mediated innate cell immune responses remains unclear. Here, a high-throughput Illumina sequencing approach was employed to determine cellular miRNA expression profiles in chicken macrophages infected with the LaSota virus, a widely used vaccine strain for mass vaccination programs against ND in poultry. Compared to the control group, 112 and 115 differentially expressed (DE) miRNAs were identified at 24 hpi (hours post inoculation) and 48 hpi, respectively. Meanwhile, 174 DE miRNAs were identified between 24 hpi and 48 hpi. Furthermore, 12 upregulated and 6 downregulated DE miRNAs were observed in common at 24 and 48 hpi compared with 0 hpi. In addition, target prediction and functional analysis of these DE miRNAs revealed significant enrichment for several signaling pathways, especially in the immune-related genes and pathways, such as the RIG-I-like receptor signaling pathway, NOD-like receptor signaling pathway, and mitogen-activated protein kinase (MAPK) signaling pathway. Our findings not only lay the foundations for further investigating the roles and regulatory mechanisms of miRNA in vaccine-mediated innate cellular immune responses, but also extend new insights into the interactions between the host and NDV infection.

## 1. Introduction

Newcastle disease (ND), caused by virulent Newcastle disease virus (NDV), an *Orthoavulavirus*, is one of the most devastating diseases that affects the poultry industry worldwide [1]. ND is listed in the World Organization for Animal Health (also named Office International des Epizooties, OIE) *Terrestrial Animal Health Code* as an economically significant pathogen for avian species and products, and any isolate from poultry infected with mesogenic and velogenic strains must be reported to the OIE [2]. NDV is a non-segmented, negative, single-stranded RNA virus with a 15 kb genome that contains six genes encoding nucleoprotein (NP), phosphoprotein (P), matrix protein (M), fusion protein (F), haemagglutinin–neuraminidase (HN), polymerase (L), and two additional non-structural proteins (V and W, that are expressed by RNA editing of the P gene) [3,4]. Depending on the severity of the clinical manifestations observed in chickens and the tropism of the virus, NDV has been classified into the following five pathotypes: Asymptomatic enteric, lentogenic, mesogenic, viscerotropic velogenic, and neurotropic velogenic [5,6]. Velogenic NDV has high virulence, mesogenic NDV has medium virulence, and lentogenic strains have low virulence or are non-virulent.

Vaccines with inactivated oil emulsion of the same viruses and live, low-virulence viruses are the most accepted prevention and control strategies for combating ND in different countries and regions around the world [6]. As is widely known, NDV infects via intranasal, oral, and ocular routes and elicits robust mucosal, cell-mediated, and humoral immune responses [7]. The well-known natural low-virulent NDV strain LaSota, which is a class II, genotype II virus [8], has been the most widely used live NDV vaccine in chickens for the last six decades and has a good stability and safety record [9]. However, the specific mechanism of the live, low-virulent vaccine strain LaSota in vaccine-mediated cellular immune responses from the sides of the innate immune cells, such as macrophages, remains unclear.

Chicken macrophages, like their mammalian counterpart, as one of the first lines of defense against microbial infection, play essential roles in both innate and adaptive immune responses [10,11,12,13,14]. Chicken macrophages are also considered to be one of the main target cells for NDV infection and growth in vivo [15]. Therefore, additional research is required to explore the functional roles of chicken macrophages in NDV vaccinations to help understand the mechanistic details of immune responses during viral infections and the ability of NDV to infect macrophages. miRNA are important post-transcriptional regulators that are widely expressed in both animal and plant species. Numerous miRNAs have been identified as being involved in the host–pathogen interaction and regulating the expression of immune genes and pathways [16]. However, the cellular miRNA profiles in chicken macrophages in response to the NDV vaccine strain LaSota infection have not been investigated. In the current study, we examined the cellular miRNA profiles in chicken macrophages in order to increase our understanding of innate cell immune responses and provide miRNA functional analysis for the immune-related pathway of the NDV vaccine strain LaSota infection. Therefore, the data presented herein have provided new viewpoints into the interactions between the host and NDV infection.

## 2. Results and Discussion

### 2.1. Preliminary Analysis of Small RNA Sequencing

**Figure 1:**
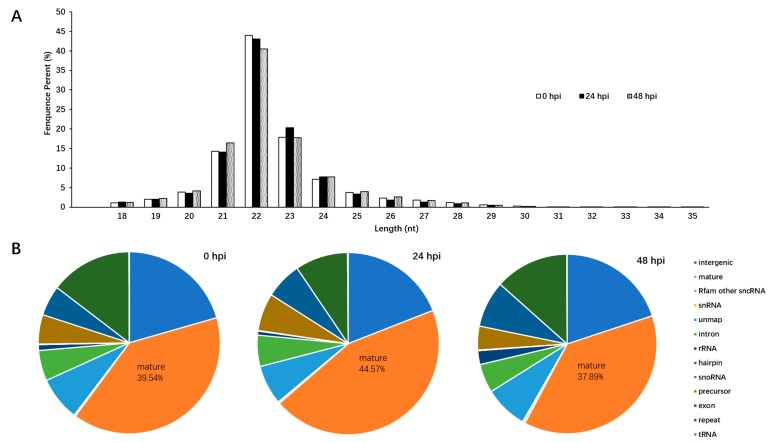
Overview of the deep sequencing length data and cellular miRNA differential expression profile in chicken macrophages infected with the LaSota strain. (**A**) Length distribution of total sRNAs in LaSota-infected at different time points and mock infected HD11 cells; (**B**) analyses of the small RNA reads generated by deep sequencing of 0 hpi, 24 hpi, and 48 hpi small RNA library.

To investigate the effects of LaSota strain infection at different time points on a cellular miRNA expression profile, three small-RNA libraries from HD11 cells infected with NDV for either 24 h post inoculation (hpi), 48 hpi, or 0 hpi were constructed and then sequenced using a deep sequencing method. In total, 53,096,137, 58,073,878, and 67,815,716 filtered high-quality reads were obtained from cells of 24 hpi, 48 hpi, and 0 hpi, respectively. Meanwhile, the majority of the small RNAs ranged between 21–23 nt in length in the three libraries; the most abundant size class was 22 nt (40.48–44%), which was consistent with the typical size range of miRNA (Figure 1A). In addition, the clean reads were aligned to repeat-associated RNAs to find matched reads and were annotated with rRNA, scRNA, snRNA, snoRNA, and tRNA from Rfam (Figure 1B). Therefore, 29,293,525 counts (39.54%) at 0 hpi, 25,662,627 counts (44.57%) at 24 hpi, and 24,021,010 counts (37.89%) at 48 hpi were annotated with mature miRNAs, respectively (Figure 1B). Among these counts, a total of 1401 mature miRNAs (including 615 known and 786 novel), 1361 mature miRNAs (including 605 known and 756 novel), and 1380 mature miRNAs (including 612 known and 768 novel) were identified at 24 hpi, 48 hpi, and 0 hpi, respectively.

### 2.2. Differentially Expressed miRNAs in Chicken Macrophages Infected with NDV Vaccine Strain LaSota

To identify miRNAs associated with NDV infection in chicken macrophages, miRNAs that were differentially expressed were identified based on a *p* value < 0.05 and a |log 2 (fold change)| > 1 as the cut-off values. As compared with 0 hpi, 112 miRNAs (including 25 known and 87 novel) were differentially expressed in cells of 24 hpi, 58 (including 15 known and 43 novel) of which were upregulated while 54 (including 10 known and 44 novel) were downregulated; 115 miRNAs (including 15 known and 100 novel) were differentially expressed in cells of 48 hpi, 76 (including 13 known and 63 novel) of which were upregulated while 39 (including 2 known and 37 novel) were downregulated. A total of 174 miRNAs were differentially expressed in cells of 48 hpi compared with 24 hpi, 97 (including 29 known and 68 novel) of which were upregulated while 77 (including 9 known and 68 novel) were downregulated. Meanwhile, 12 upregulated and 6 downregulated differentially expressed (DE) miRNAs were observed in common at 24 and 48 hpi compared with 0 hpi (Table 1). In addition, we found that at 24 hpi and 48 hpi, 46 and 64 DE miRNAs were uniquely upregulated, and 48 and 33 were uniquely downregulated compared with 0 hpi, respectively. Therefore, these findings suggested that different miRNA expression characteristics exist among the NDV vaccine strain LaSota at different post-infection times in chicken macrophages.

To validate the sequencing data, six DE miRNAs (including three upregulated and three downregulated miRNAs) were selected for qRT-PCR analysis. The results confirmed the upregulation of expression of three miRNAs (gga-novel-800-mature, gga-novel-862-mature, and gga-miR-6606-5p) and the downregulation of expression of three miRNAs (gga-novel-880-mature, gga-novel-20-mature, and gga-novel-892-mature) in NDV-infected cells as compared with the mock infected cells. Thus, the expression profiles obtained from the deep sequencing method were reliable and suitable for use in further analysis.

### 2.3. Target Gene Prediction and Functional Annotation Analyses for DE miRNAs after NDV Infection in Chicken Macrophages

To understand the molecular functions and biological processes of the DE miRNAs detailed in this work, two independent algorithms, RNAhybrid and miRanda, were used to predict the mRNA targets for each of the miRNAs that were significantly differentially expressed. As a result, a total of 7506 unique putative genes were targeted by 97 DE miRNAs between cells at 24 hpi and 0 hpi (Table S1), 8539 unique putative genes were targeted by 108 DE miRNAs between cells at 48 hpi and 0 hpi (Table S2), and 8306 unique putative genes were targeted by 121 DE miRNAs between cells at 48 hpi and 24 hpi (Table S3). Our results, in agreement with those of other investigators [16,17], show that a single miRNA molecule can regulate multiple genes. Conversely, multiple miRNAs can regulate a single target gene, indicating that the miRNA-based regulatory network is extremely complicated.

**Figure 2:**
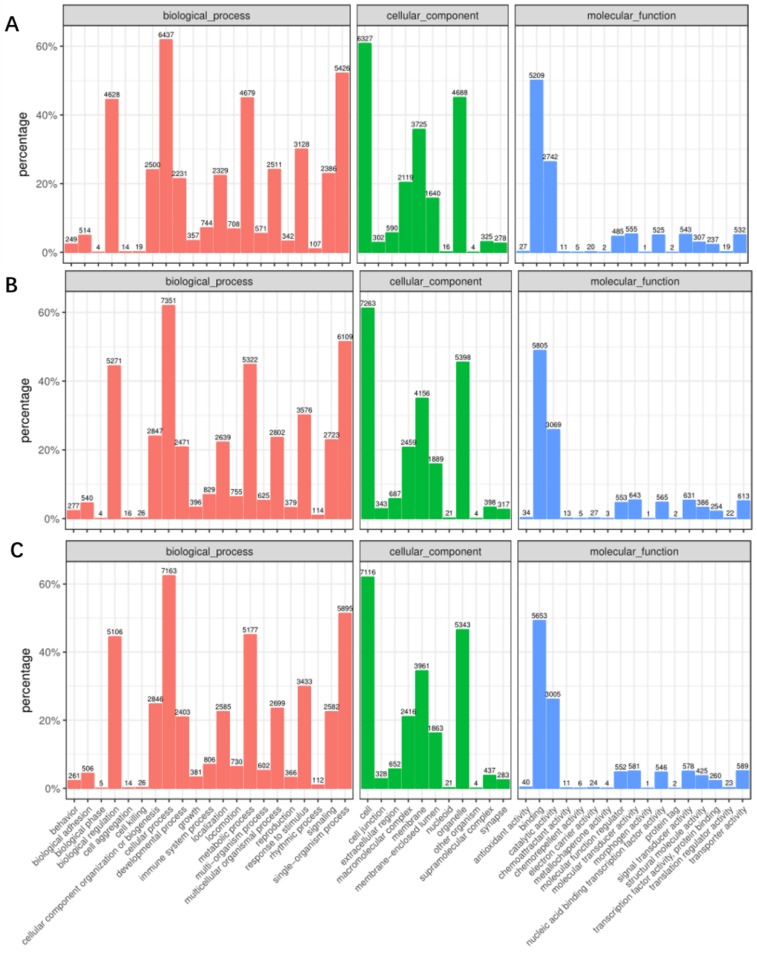
Gene ontology (GO) classification of miRNA-targeted mRNAs into three categories: Molecular function, cell component, and biological process between cells at 24 hpi and 0 hpi (**A**); between the cells at 48 hpi and 0 hpi (**B**); and between the cells at 48 hpi and 24 hpi (**C**).

Next, these mRNA targets were sorted by the enrichment of GO (gene ontology) categories and mainly clustered into different functional groups. The distribution of GO terms in the biological process, cellular component, and molecular function among 0 hpi, 24 hpi, and 48 hpi as shown in Figure 2. We found that, in all three libraries, the biological processes with the highest target numbers were cellular processes (62%) (GO:0009987), single-organism processes (52%) (GO:0044699), metabolic processes (45%) (GO:0008152), and biological regulation (45%) (GO:0065007); cell (61%) (GO:0005623), organelle (45%) (GO:0043226), membrane (36%) (GO:0016020), and macromolecular complexes (20%) (GO:0032991) were among the most abundant classes in the molecular function category; the common cellular component terms were binding (50%) (GO:0005488), catalytic activity (26%) (GO:0003824), molecular transducer activity (5%) (GO:0060089), and signal transducer activity (5%) (GO:0004871) (Table S4).

**Figure 3:**
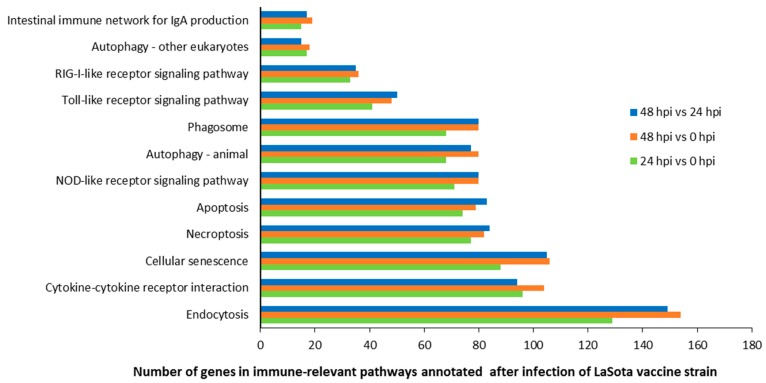
Number of genes in 12 immune-relevant pathways annotated after infection with the LaSota vaccine strain.

Meanwhile, these targets were functionally analyzed by KEGG (Kyoto Encyclopedia of Genes Genomes) pathway annotation; 162, 161, and 162 KEGG pathways were obtained for the groups of 24 hpi vs. 0 hpi, 48 hpi vs. 0 hpi, and 48 hpi vs. 24 hpi. It was evident from our observations that a large number of enriched genes were mainly involved in the mitogen-activated protein kinase (MAPK) signaling pathway (gga04010), focal adhesion (gga04510), mTOR signaling pathway (gga04150), and herpes simplex infection (gga05168), which were observed between cells at 24 hpi and 0 hpi, while the most abundant KEGG terms corresponded to the MAPK signaling pathway, focal adhesion, protein processing in endoplasmic reticulum (gga04141), and cellular senescence (gga04218), which were present between cells at 48 hpi and 0 hpi. Moreover, the most abundant of the KEGG terms corresponded to the MAPK signaling pathway, focal adhesion, tight junction (gga04530), cellular senescence, and protein processing in endoplasmic reticulum were obtained between cells at 48 hpi and 24 hpi. As we observed, at least 15 KEGG pathways, including 2714, 3086, and 2999 genes were shown to be closely related to immune responses in the groups of 24 hpi vs. 0 hpi, 48 hpi vs. 0 hpi, and 48 hpi vs. 24 hpi (Figure 3).

**Figure 4:**
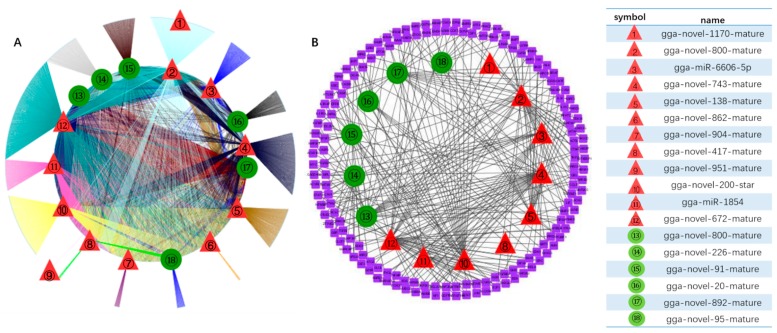
DE miRNAs targets regulatory genes in pathways between samples infected with LaSota at different time points. (**A**) 18 common DE miRNA-targets regulatory networks including 162 and 161 Kyoto Encyclopedia of Genes Genomes (KEGG) pathways in groups of 24 hpi vs 0 hpi and 48 hpi vs 0 hpi; (**B**) nine immune-related pathways were selected to explore the targeting relationship between 15 of 18 DE miRNAs and immune factors. The red triangle represents up-regulated miRNAs and the green circle represents down-regulated miRNAs.

**Figure 5:**
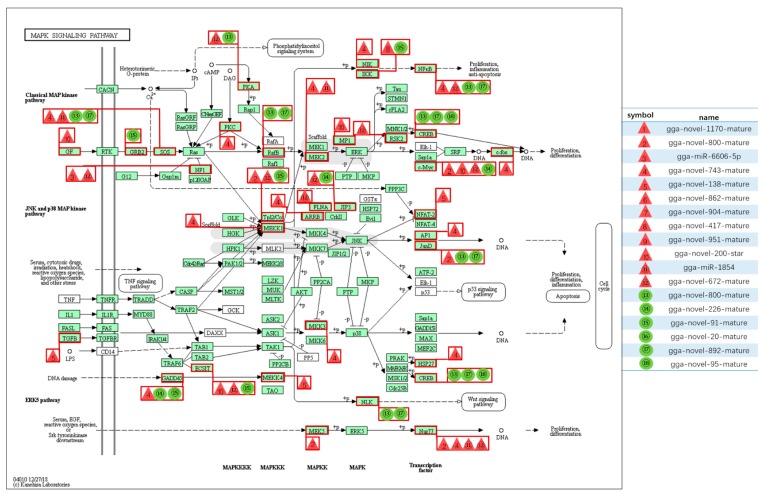
The mitogen-activated protein kinase (MAPK) signaling pathway and related miRNAs. The red triangle represents up-regulated miRNAs and the green circle represents down-regulated miRNAs.

Among all the DE miRNAs, there are 18 DE miRNAs that remain up-/down-regulated when infected with the LaSota vaccine strain even as the infection time progresses. A total of 7181 unique putative genes were targeted by 18 DE miRNAs between cells infected with NDV and mock infected cells (Figure 4A and Table S5), at least 1485 of which were immune-relevant genes and were involved in immune-related pathways (Figure 4B and Table S6), such as cellular senescence, the cytokine–cytokine receptor interaction, the MAPK signaling pathway, RIG-I-like receptor signaling pathway, apoptosis, necroptosis, the NOD-like receptor signaling pathway, Toll-like receptor signaling pathway, and endocytosis. For example, the MAPK signaling pathway, which is involved in both innate and adaptive immune responses [18,19], was represented by 120 genes and contained at least four distinctly regulated groups of MAPKs, Jun amino-terminal kinases (JNK1/2/3), extracellular signal-related kinases (ERK)-1/2, ERK5, and p38 proteins (p38 alpha/beta/gamma/delta) (Figure 5 and Table S7). As was observed, the central proteins, MEKK1, MEKK4, MEK2, and c-Myc, within the MAPK signaling pathway were regulated by gga-novel-743-mature, gga-novel-138-mature, gga-miR-1584, gga-novel-800-mature, gga-novel-200-star, gga-novel-672-mature, and gga-novel-226-mature.

Although mitochondrial antiviral-signaling protein (MAVS; also known as IPS-1, VISA, Cardif) is a central adaptor protein in retinoic acid-inducible gene I (RIG-I)-mediated antiviral innate immunity in mammals and birds (for example, goose MAVS is a vital antiviral that acts by activating type I interferon when infected with NDV virulent strain Herts33/56 [20]), very little is known about the mechanisms for miRNA regulation of type I interferon induction in NDV infection. In the present study, we found that the essential adaptor protein MAVS was annotated as a target by downregulated gga-miR-6609-3p, gga-novel-103-mature, and gga-novel-328-mature. Meanwhile, a previous study suggested that NDV can activate the NOD-like receptor protein 3 (NLRP3) inflammasome [21], a critical contributor in inflammatory responses; however, the detailed mechanism remains unknown. In this study, NLRP3 was targeted by upregulated DE miRNA, such as gga-novel-200-star, gga-novel-36-star, and gga-novel-424-mature. However, identification of DE cellular miRNA is just the first step towards understanding miRNA regulation of interactions between the host and viral infection. Then, more works were planned to uncover the underlying mechanisms of how these potential genes are targeted by DE miRNA and their roles in the live, low-virulent vaccine strain LaSota mediated immune cell responses from the sides of host innate immune cells, such as chicken macrophages in the current study.

A previous study demonstrated that 61 DE miRNAs (including 36 upregulated and 25 downregulated miRNAs) were observed in visceral tissues from LaSota-infected specific-pathogen-free (SPF) chicken embryos for 36 h as compared to the mock infected sample [22]; however, a much greater number of DE miRNA (112 and 115 DE miRNA at 24 hpi and 48 hpi) were obtained in the chicken macrophage, an innate immune cell, and target cell for NDV infection in vivo. Therefore, the finding presented here also revealed that different miRNA regulation profiles exist among different cell lines infected with the same virus.

## 3. Material and Methods

### 3.1. In Vitro Infection of HD11 Cells with LaSota

NDV vaccine strain LaSota (lentogenic, genotype II within class II, GenBank: AF077761) was purchased from ATCC. The virus was propagated in 9–11-day-old SPF chicken embryos (MERIAL, Beijing, China), and was purified directly from the infected allantois fluid, as described in previous studies [10,23]. Chicken-origin macrophage HD11, a permanent chicken bone marrow macrophages cell line was used in numerous chicken innate immune cell models for avian viruses, including the NDV vaccine strain LaSota [13], and was kindly gifted by Prof. Daxin Peng, Yangzhou University. The HD11 cells were cultured in DMEM/F-12 (Dulbecco’s Modified Eagle Medium/Nutrient Mixture F-12) supplemented with 10% fetal bovine serum (FBS) (Gibco, Shanghai, China) and 1% antibiotic (100 µg/mL streptomycin and 100 U/mL penicillin) (Gibco, Shanghai, China) at 37 °C with 5% CO_2_. When the number of cells grew by 80%, the cell plates were washed twice with phosphate-buffered saline (PBS). Afterward, the cells were incubated for 2 h with LaSota at 2 multiplicity of infection (MOI) and control groups were set up with DMEM serum-free medium. There were 3 set replicates per group, respectively. After 2 h, the cells were washed twice with PBS, and then 1% antibiotic and 2% FBS DMEM medium was added to each well and cultured at 37 °C Total RNA was extracted at 24 hpi and 48 hpi, respectively. Each group was processed in triplicate and pooled separately for subsequent total RNA extraction. Then, three independent repeats of each time point were performed for deep sequencing.

### 3.2. RNA Extraction

Total RNA was extracted using Tripure (Roche, Mannheim, Germany) following the manufacturer’s recommendations. The quantity and purity of RNA were determined by ND-1000 Nanodrop^®^ Spectrophotometer (Thermo Scientific, Waltham, MA, USA), at A260/A280 nm ≥ 2, and the quality and concentration of RNA were determined by an Agilent 2100 Bioanalyzer. Only samples with an RNA integrity number greater than 8 were used for sequencing.

### 3.3. Small RNA Library Preparation and Illumina Sequencing

An amount of 2 μg total RNA per sample was used as input for small RNA library preparation. The total RNA from each sample was sequentially ligated to 3′ and 5′ small RNA adapters using T4 RNA ligase according the manufacturer’s instructions (Illumina^®^ TruSeq^®^ Small RNA Library Prep Kit, Illumina). Next, cDNAs were synthesized through reverse transcription using M-MLV reverse transcriptase and the cDNAs were amplified by PCR. Then, 18–30 nt fragments of total small RNA from PCR products were isolated using PAGE. Subsequently, three small RNA libraries, which included two NDV-infected samples and one mock infected sample, were sequenced on an Illumina HiSeq 2500 platform (Illumina, San Diego, CA, USA).

### 3.4. Quantitative Real-Time RT-PCR (qRT-PCR)

To validate the small RNA sequencing data, qRT-PCR was performed to validate the expression profile of selected DE miRNAs using Bulge-loop™ miRNA qRT-PCR Primer Sets (one RT primer and a pair of qPCR primers for each set) and performed on an ABI StepOne System (Applied Biosystems, Warrington, UK). The gene U6 was used as the reference gene. The relative expression level of each miRNA in the infected samples was calculated using the 2^−ΔΔCt^ method [24].

### 3.5. Sequencing Data Analysis

Sequences that match the miRBase database are known as mature miRNA and sequences that do not match the database are novel miRNA sequences. miRNAs with a *p* value ≤ 0.05 and log2 Fold Change (Log2 FC) ≥ 1 are defined as DE miRNAs. Meanwhile, the miRNA target prediction software, RNAhybrid and miRanda, were used to predict the potential targets of DE miRNAs. To determine the primary function of predicted target genes regulated by DE miRNAs using GO and KEGG functional classifications. Furthermore, the network regulated by DE miRNAs was visualized using the Cytoscape software. The red triangle represents up-regulated miRNAs and the green circle represents down-regulated miRNAs.

## 4. Conclusions

In conclusion, we identified miRNA and demonstrated the characterization of cellular miRNA in chicken macrophages infected with the NDV vaccine strain LaSota using deep sequencing methods. Sixteen novel DE miRNA (including ten upregulated and six downregulated DE miRNA, respectively) and two known upregulated miRNA candidates were observed in common at 24 hpi and 48 hpi compared with 0 hpi. In addition, the number of potential target genes related to immune-relevant genes and pathways were obtained by GO enrichment and KEGG pathway analysis. Taken together, while these results identify the DE miRNA profiles in chicken macrophages infected with the NDV vaccine strain LaSota, further studies are needed to understand how the live, low-virulent vaccine strain LaSota mediated innate immune responses from the side of chicken macrophages.

## Figures and Tables

**Table 1:** Detailed information on 18 common differentially expressed (DE) miRNAs that were up-/down-regulated in groups of 24 h post inoculation (hpi) vs. 0 hpi and 48 hpi vs. 0 hpi detected by deep sequencing methods.

Number	Id	Sequences (5′–3′)	Regulated	24 hpi vs. 0 hpi		48 hpi vs. 0 hpi	
				Log2 FC	*p* Value	Log2 FC	*p* Value
1	gga-novel-1170-mature	UAUGGGAGGAACUGAAUGACAUG	up	2.05	1.24 × 10^−2^	1.82	1.63 × 10^−2^
2	gga-novel-800-mature	UGGGCGGCUGCGGGAGGG	up	3.76	1.27 × 10^−3^	3.10	2.11 × 10^−2^
3	gga-miR-6606-5p	GAGGAGCGGGAGGAGCGGGA	up	1.45	1.36 × 10^−2^	1.53	7.19 × 10^−3^
4	gga-novel-743-mature	UGGGGGUGCAGGUGGGGGGCU	up	1.68	4.81 × 10^−2^	2.34	4.74 × 10^−3^
5	gga-novel-138-mature	ACGGGACGGGGCGGGACGGCGC	up	1.36	3.31 × 10^−2^	1.20	4.87 × 10^−2^
6	gga-novel-862-mature	GAGCAAGGUACGGGGGGGU	up	2.32	3.27 × 10^−4^	2.09	1.00 × 10^−3^
7	gga-novel-904-mature	AGCAGAGAGAAGGGAUGAGGCU	up	1.57	1.16 × 10^−2^	1.24	4.15 × 10^−2^
8	gga-novel-417-mature	UGCUGGUAGGGGCCGACGACC	up	2.46	9.16 × 10^−3^	2.49	1.15 × 10^−2^
9	gga-novel-951-mature	AUGGAGGCGUGGGUUUUU	up	3.21	9.57 × 10^−6^	3.50	3.59 × 10^−7^
10	gga-novel-200-star	UGGGGAGGCCGCAGUGCAGGGCAA	up	2.99	1.02 × 10^−2^	2.63	2.22 × 10^−2^
11	gga-miR-1584	CCGGGUGGGGCUGGGCUGGG	up	2.36	9.62 × 10^−3^	3.22	1.10 × 10^−4^
12	gga-novel-672-mature	CCGCGGGGUGGGCGGGGGGCG	up	2.10	7.98 × 10^−3^	2.44	6.66 × 10^−4^
13	gga-novel-880-mature	UGCCGCUGCCCGGUGCUCACACU	down	−3.79	2.28 × 10^−3^	−2.84	1.14 × 10^−2^
14	gga-novel-226-mature	CGCAGCUCCGUUCCGUCCCCG	down	−1.62	2.75 × 10^−2^	−1.41	4.23 × 10^−2^
15	gga-novel-91-mature	UCCGCAGCUCCACUCCUGUCAC	down	−1.25	4.03 × 10^−2^	−2.23	3.67 × 10^−4^
16	gga-novel-20-mature	GCUCCUGCCUGGCUCGCCA	down	−3.64	1.71 × 10^−8^	−1.31	3.19 × 10^−2^
17	gga-novel-892-mature	UGCCGCUGCCCGGUGCUCACACU	down	−3.79	2.28 × 10^−3^	−2.84	1.14 × 10^−2^
18	gga-novel-95-mature	CUGCACUGCCACGCCGCGUUCC	down	−1.30	2.07 × 10^−2^	−1.13	4.48 × 10^−2^

## Supplementary Materials

The following are available online at https://www.mdpi.com/2076-0817/8/3/123/s1. Table S1 List for targets of all DE miRNAs of 24 hpi vs. 0 hpi; Table S2 List for targets of all DE miRNAs of 48 hpi vs. 0 hpi; Table S3 List for targets of all DE miRNAs of 48 hpi vs. 24 hpi; Table S4 GO classification for targets of all DE miRNAs; Table S5 Detailed information on 18 DE miRNAs and their corresponding target genes; Table S6 Detailed information on 18 DE miRNAs and their corresponding immune-related target genes and pathways; Table S7 Detailed information on MAPK signaling pathway and related DE miRNAs.
